# Artificial grammar learning meets formal language theory: an overview

**DOI:** 10.1098/rstb.2012.0103

**Published:** 2012-07-19

**Authors:** W. Tecumseh Fitch, Angela D. Friederici

**Affiliations:** 1 Department of Cognitive Biology, University of Vienna, Althanstrasse 14, Vienna 1090, Austria; 2 Max Planck Institute for Human Cognitive and Brain Sciences, Stephanstrasse 1a, 04103 Leipzig, Germany

**Keywords:** artificial grammar learning, formal language theory, comparative neuroscience, neurolinguistics

## Abstract

Formal language theory (FLT), part of the broader mathematical theory of computation, provides a systematic terminology and set of conventions for describing rules and the structures they generate, along with a rich body of discoveries and theorems concerning generative rule systems. Despite its name, FLT is not limited to human language, but is equally applicable to computer programs, music, visual patterns, animal vocalizations, RNA structure and even dance. In the last decade, this theory has been profitably used to frame hypotheses and to design brain imaging and animal-learning experiments, mostly using the ‘artificial grammar-learning’ paradigm. We offer a brief, non-technical introduction to FLT and then a more detailed analysis of empirical research based on this theory. We suggest that progress has been hampered by a pervasive conflation of distinct issues, including hierarchy, dependency, complexity and recursion. We offer clarifications of several relevant hypotheses and the experimental designs necessary to test them. We finally review the recent brain imaging literature, using formal languages, identifying areas of convergence and outstanding debates. We conclude that FLT has much to offer scientists who are interested in rigorous empirical investigations of human cognition from a neuroscientific and comparative perspective.

## 1. Introduction

Formal language theory (FLT) has its roots in mathematics [1,2] but was established in its modern form by Noam Chomsky in an attempt to systematically investigate the computational basis of human language [3,4]. Since these beginnings, the theory has been continually expanded to cover other scientific domains. The most prominent new application was in computer science, where the study of FLT is now a core part of the standard curriculum, providing the theoretical foundation for fundamental issues such as programming language structure and compiler design [5]. Psychologists have used FLT to explore learning and pattern-processing abilities in humans and other species [6–10], while in neuroscience the theory has been used in neuroimaging experiments to better understand the neural computations of hierarchy and sequence [11,12]. Finally, in biology, FLT has been used to analyse diverse topics such as the structure of RNA molecules [13,14] and the sequential structure of chickadee song [15]. FLT has thus grown far beyond its original roots in language, to become a key component of the theory of computation, applicable to virtually any rule-governed system, in any domain.

In this paper, we review recent progress in applying FLT to empirical research in animal cognition and neuroscience, as well as highlighting some pitfalls that can accompany attempts to merge theory and practice. We start with a non-technical overview of FLT, intended to give an intuitive understanding of the theory and its significance and to provide a gentle preparatory overview for the more rigorous paper by Jaeger & Rogers [16]. We then provide a more detailed analysis of the difficulties involved in translating this body of theory into an empirical research programme. We start with the difficulties caused by the use of infinity as a tool for proofs in mathematics, which leaves such proofs technically irrelevant in the real world of finite brains and finite time. We provide a detailed analysis of one particular rule system, the so-called ‘A*^n^*B*^n^* grammar’, which has been employed in many recent studies, both in neuroscience and in animal cognition. We suggest that this grammar is appropriate for answering certain interesting questions, but has sometimes been over-extended to address issues for which it is poorly suited, for which we suggest alternative, more appropriate grammars. In the process, we highlight the need to clearly distinguish among a number of separate issues, which—although related—should not be conflated. These include notions such as hierarchical structure versus centre-embedding, context-freeness versus long-distance dependency and formal complexity versus recursion. FLT provides the theoretical concepts and terminology to clearly distinguish among all of these terms, and we argue that it should be used to do so more rigorously in the future. We then provide a detailed look at some of the recent brain imaging literature using FLT, highlighting the areas of nascent agreement along with outstanding open questions. We conclude by pointing out some areas within FLT that remain little explored, but might provide fertile ground for future research.

## 2. Formal language theory and the theory of computation

We have an intuitive sense that some cognitive computations are more difficult than others. For most people, it is harder to play chess or solve equations than to buy groceries or drive a car. For most of us, it is more difficult to parse sentences in a non-native language (regardless of our level of proficiency) than in our native language. However, a central finding of computer science is that our intuitions about complexity do not necessarily apply to computer programs. In fact, it has proved relatively easy to create machines that can play chess at a high level, but so far impossible to create adequate car-driving systems. Because of this, an important component of modern computer science is a framework for quantifying the ‘difficulty’ or ‘complexity’ of a computational problem or algorithm in terms that are explicit and unambiguous. Starting with the work of the brilliant mathematician Alan Turing, and combined with further insights owing to Gödel, Church, Post, Kleene, Chomsky and many others, FLT has grown today into one key pillar of the theory of computation (and thus compiler design and many other aspects of computer science). The other main pillars are the theory of computability (what problems can or cannot be solved) and the theory of problem complexity (how the difficulty of problems scales with their size) [5,17].

The theory of computation provides the practical basis for software tools we use everyday, which thus provide useful illustrations of the core concepts of FLT. We favour such everyday examples from computers, rather than mathematical formalisms, because we expect that most of our readers will have some experience with the former but not necessarily with the latter. More detailed and mathematical treatments are easy to find [5,17–20], and a paper in this issue provides a particularly accessible formal introduction designed for experimentalists [16].

### (a) Regular expressions

We start our survey with a simple, well-defined computational system, termed a finite-state machine, which is equivalent to another simple construct called a ‘regular expression’. Search functions, such as the *dir* command in DOS, or the *ls* function in UNIX, provide everyday examples. Such functions use a syntax that allows us to search for the arbitrary target pattern in a large database of words and/or numbers. Given a set of file names:

> filenames = {a.wav, b.doc, c.bmp, MySong.doc, MySong.wav}

running the function:

> ls *.wav filenames

(or the equivalent with dir in DOS) on this set will return the subset

> {a.wav, MySong.wav}.

The search string ‘*.wav’ says, in effect, ‘give me all the strings that end with “.wav” ’. The * character tells the *ls* or *dir* command that the string(s) can start with any characters in the alphabet, we do not care which or how many. This search string is one simple example of a general framework called ‘*regular expressions*’, which provide a very powerful basis for computer-based search that underlies searching, replacing and other functions in many computer programs. This ability to use regular expressions to match patterns was first instantiated in the *grep* function in UNIX/Linux, and has proved so useful that the term ‘grep’ has entered hacker lingo as a verb meaning ‘to search by computer’.

Regular expressions are composed using a few simple but powerful rules and operators, familiar to many computer users. The operator *, as used earlier, means ‘any string of any length’ and by appending it to our search string (e.g. ‘string’), we can find our target pattern even if preceded by anything (*string), followed by anything (string*) or buried in anything (*string*).^1^ More specific operators also allow us to specify a single, unspecified character (?), a character from a particular set (e.g. numbers {0–9} versus letters {a–z}), or even a specific number of characters from a certain set. Any time you have some pattern or a set of patterns that can be captured by a regular expression, you can use *grep* to search an arbitrary database for that pattern. You can *grep* for your name or email address or telephone number in the archives of a discussion group, or *grep* for a particular gene sequence in the online human genome database. The search engine Google is an extended version of *grep* that takes the entire web as its database. Regular expressions are at the core of computer search in today's world.

Given this flexibility and power, we might think that regular expressions are capable of specifying any kind of pattern that we can imagine. Crucially, however, this turns out not to be true. For instance, imagine a simple symmetrical pattern where a particular number of items of type A is followed by the same number of a different type B. Examples of this set include {AB, AABB, AAABBB, AAAABBBB, etc.}, and extend indefinitely (so a string of 1346 ‘A's followed by 1346 ‘B's is still a member of the set). This pattern is notated A*^n^*B*^n^* in FLT. It is easily proved that this set cannot be specified by a regular expression (see the textbooks listed earlier for mathematical proofs). We conclude from this fact that there are patterns that we can conceive of, and that we could easily (if laboriously) recognize ourselves, but that cannot be captured by a regular expression. Why does this matter? Because, as demonstrated by the mathematician Stephen Kleene in 1956 in the theorem that bears his name, regular expressions and the corresponding rule sets termed ‘regular grammars’ are exactly equivalent to one of the most ubiquitous classes of computing devices, which are termed ‘finite-state automata’ [21].

### (b) Finite-state automata

FLT relies on abstract models of computational systems termed ‘automata’ (and often, perhaps confusingly, also often called ‘machines’). Two canonical examples of such models are the finite-state automaton (FSA) and the Turing machine. Automata such as these are mathematical abstractions, not real devices designed to be manufactured. For example, the Turing machine includes as part of its mathematical definition a storage tape of infinite length, and thus we could never build a real Turing machine. Many automata, although well-defined in theory, are unbuildable in practice (a fact that has some implications that we will discuss later). Despite this, the abstract notion of a Turing machine is extremely important and powerful in mathematics and FLT: infinity is a powerful tool for mathematical abstraction, but not a real thing that we find in the world.

The simplest class of well-defined automata are called finite-state automata because they have a finite number of operating states or ‘positions’. The FSA starts at a predefined start state, and then jumps between its other states, depending only on its current input and current state. For each of these jumps, it can emit an output symbol as it hops along. Thus, an FSA can be fully defined by its set of states, its input alphabet (the input symbols that it recognizes), an optional output alphabet (which might or might not be different) and a function that tells it which is the next state to go to, given its current state and current input. Any given FSA is capable of ‘recognizing’ a certain set of patterns, and rejecting others. By ‘recognize’ we mean simply that, given this pattern as input, it can generate some particular prespecified output (e.g. ‘OK’). The set of patterns recognizable by an FSA may be infinite. Because of this, and its simplicity, the FSA is a good starting point for further discussions of automata and computational complexity. Critically, as already mentioned, Kleene's Theorem demonstrates the equivalence, or interchangeability, of FSAs and regular expressions.

**Figure 1.**
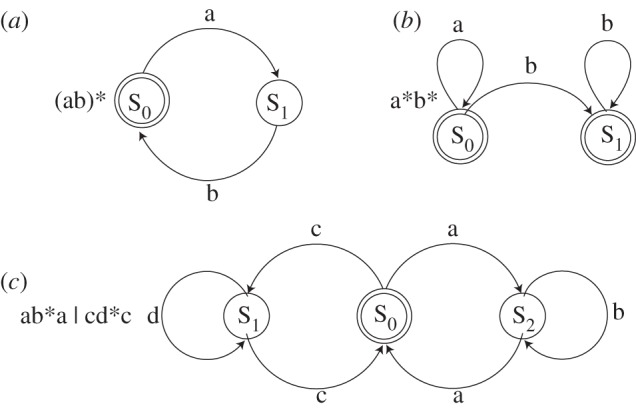
Three examples of simple finite-state automata and their stringsets. Circles represent states, arcs represent transitions between states, with the corresponding symbols, and double circles indicate ‘accept’ states. (*a*) The (ab)* or (ab)*^n^*: accepts strings of zero or more ‘ab’ bigrams. (*b*) The a*b*: accepts any number of ‘a's followed by any number of ‘b's. (*c*) A long-distance dependency: this automaton illustrates that FSAs can also check for arbitrarily long-distance dependencies. This grammar accepts strings of the form ab*a, where any number of ‘b's can intervene between the two ‘dependent’ ‘a's, (or similarly for cd*c strings).

One point, overlooked by many, is that FSAs can recognize (or generate) a simple, long-distance dependency of the start-and-end sort (e.g. ab*a or cd*c) as the automaton in figure 1*c* shows (p. 1103). However, other patterns are clearly beyond the capabilities of an FSA, because we already know that certain patterns, such as A*^n^*B*^n^*, cannot be captured by regular expressions: they are beyond the capabilities of our simplest class of automaton. Thus, something with more computational power is clearly needed.

### (c) Turing machines

Other automata take an FSA as their starting point, and achieve additional computational power by adding some additional form of memory. The most important and powerful such automaton is the Turing machine, which adds to an FSA (the ‘controller’) a storage tape of unbounded length, on which symbols can be written or erased.^2^ Thus, in addition to possessing a large (but finite) set of states that its controller can occupy at any one moment, the Turing machine has by virtue of this tape an additional unlimited form of memory for storing past operations, intermediate results and so on. A Turing machine can easily recognize the A*^n^*B*^n^* language described earlier: it simply stores the number of times A has been repeated (that is, writes successive integers every time its input jumps from A to A) and then compares that with the number of Bs (B to B jumps). Thus, Turing machines are more ‘powerful’ than their FSA component, in the sense that they can recognize patterns and solve problems unsolvable by any FSA. This is not surprising given their additional resources. What is altogether more remarkable is that the Turing machine is capable of computing ANY deterministic function whatsoever: if something is computable, a Turing machine can compute it. Thus, modern computer scientists accept the Turing machine as their very definition of ‘computability’, broadly accepting the ‘Church/Turing thesis’ that a function is computable if and only if it is computable by a Turing machine.

The Turing machine and FSA are well-defined automata that provide useful endpoints for a scale of *computational power*: the FSA provides the lower level (which is powerful and practically useful, but has its limits), while the Turing machine provides the upper limit (it is all-powerful in the sense that, if a function is computable at all, an automaton in this class can compute it). We will use the term ‘computational power’ in this paper in this specific sense, framed by the specific automata discussed in FLT. We do not mean to imply by this that this is the only way to insightfully characterize the power of algorithms, or that ultimately this is the best way to think about the different aspects of brain function. What this specific sense of computational power gives us is an explicit, formal axis along which any particular algorithm can be placed, which thus provides one useful dimension along which to characterize the rule-governed capacities of a machine, or a human or animal subject. Other potentially useful dimensions will be discussed briefly at the end of this paper.

Given these two endpoints, we might immediately ask two questions:

— Are there other intermediate classes of automata, with powers greater than an FSA but less than a Turing machine?
— Where do human computational powers (or those of other species) fall along this spectrum?

### (d) The Chomsky hierarchy

In an attempt to answer the second question, the young Noam Chomsky and his colleagues built upon the framework already discussed and provided a positive answer to the first question. Chomsky outlined a set of intermediate formal possibilities, between the extremes of Turing machines and finite-state automata, and arranged them in a theoretical hierarchy that now bears his name. Because both the nature and the importance of this hierarchy are sometimes misunderstood, we will try to make clear here both what the Chomsky hierarchy is, and why it is important. First, let us consider the relationship between a Turing machine and an FSA. Because every Turing machine contains within it an FSA, anything computable (e.g. any pattern that can be recognized) by an FSA is perforce computable by a Turing machine. Thus, the set of FSA-recognizable patterns is a proper subset of those computable by a Turing machine. This is obvious from the way in which these automata are defined.

**Figure 2.**
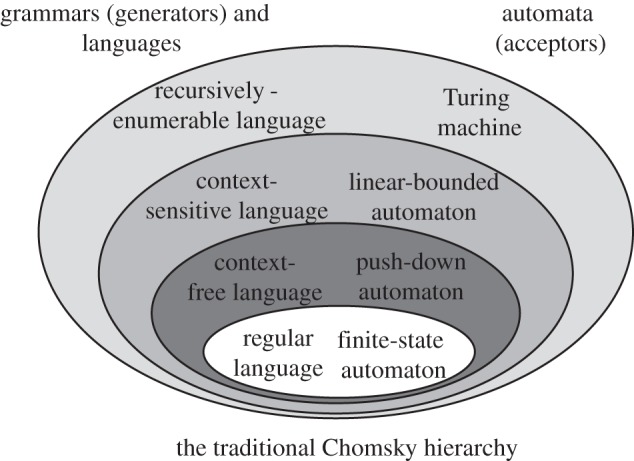
The Chomsky hierarchy for grammars, languages and automata. The Chomsky hierarchy is an inclusion hierarchy that aligns particular forms of grammar, and the languages they generate, with particular classes of automata—abstract computational ‘machines’ that can be constructed to accept such languages. All of the grey circles beyond the innermost white circle represent the *supra-regular* grammars and languages, which require computational power above the level of a finite-state automaton. See Jäger & Rogers [16] for more details.

The Chomsky hierarchy incorporates several intermediate levels of automata, which have in common with the Turing machine an additional memory system but discard the assumption that this memory can be freely accessed. For example, a ‘pushdown automaton’ (PDA) includes an FSA and a pushdown stack (which is a memory that can only return the most recent item placed upon it, like the stack of trays in a cafeteria). Because a stack is more limited than the infinite tape, it is intuitive (and can be shown mathematically) that the PDA is less powerful than a Turing machine, while being more powerful than an FSA. And as before, the set of patterns recognizable by the PDA is a proper subset of those captured by the Turing machine. In fact, a PDA can recognize the A*^n^*B*^n^* language discussed earlier, which is beyond an FSA. Thus, we now have a nested set of patterns, enclosed one within the other like Russian dolls. A second intermediate form of automaton is called a linear-bounded automaton and includes both the FSA and PDA within it. Figure 2 provides an illustration of this nested hierarchy.

The Chomsky hierarchy provides a broad framework for discussing the computational power of automata, universally accepted in this role in theoretical computer science, algorithmic theory, FLT and discrete mathematics. It is well-defined, explicit and unambiguous and discussed and defined in any textbook on these topics. The Chomsky hierarchy contains no hidden assumptions about Universal Grammar, the ‘poverty of the stimulus’ argument or other of Chomsky's various more controversial ideas about language. A computer scientist using the Chomsky hierarchy need not accept other aspects of Chomsky's thought, any more than a logician using Bertrand Russell's innovations in mathematical logic need accept Russell's pacifism or atheism. While this should be an obvious point, we have been surprised by how often the distinction gets blurred. Although there are other ways to arrange automata in hierarchies of ascending power, as well as finer subdivisions of existing hierarchies [16], we focus on the Chomsky hierarchy in this work owing to its interdisciplinary acceptance and understanding. Anyone who studies basic computer science or the theory of algorithms will be familiar with the framework, and this is more than can be said for any other framework we know of. We do not claim that this whole set of automata (including FSAs and Turing machines) is the best or the most insightful way of arranging the different types of neural computations that we ultimately want to understand as psychologists, biologists or neuroscientists [22]. Indeed, as our understanding of neural computation in vertebrates progresses, it seems likely that different hierarchies will arise, and prove to be more useful. Until such progress is made, however, FLT seems to provide the best theoretical starting point, and indeed has no obvious competition.

One might object that some models of neural computation, especially connectionist networks, offer just such an alternative framework. This is not true. In fact, neural networks are automata, like any other well-defined computational system. Indeed, surprisingly simple recurrent neural networks can be shown to be Turing-equivalent in principle [23] (although programming such networks to do some of the tasks they could perform in theory has been difficult or impossible in practice). Other classes of automata, such as augmented transition networks, also take their place within this scheme. The power of the Turing machine framework is that it includes *any* computational device: the definition is formally equivalent to a vast number of alternative implementations, in a wide variety of forms (indeed many different computational frameworks were initially offered as alternatives to Turing machines, but were later shown to be Turing-equivalent). Thus, the term ‘computation’ is used here, and in computer science in general, in a very broad and inclusive sense to capture any algorithm (information processing system). Neural networks and a vast array of other implementations are part of this classification, not alternatives to it.

### (e) Formal language theory

With these preliminaries behind us, and the basic aims and principles of computational theory clarified, we can now introduce the terminology and principles of FLT. On the one hand, fortunately, this terminology is simple and unambiguous, and thus quite familiar because it maps the core technical concepts onto everyday terms. On the other hand, this familiarity can be deceptive and misleading. As typical with technical terms, we must beware of unwittingly slipping from interpreting the words in their technical sense to their broader everyday sense.

First, the terminology: an *alphabet* A is a set containing a finite number of indivisible symbols. A *string* (often termed, more confusingly, a *word* or a *sentence*) is a finite sequence of symbols, and a *string-over-A* is a finite sequence of symbols from A. The set of all such strings-over-A is denoted A*, pronounced ‘A star’.^3^ Finally, a *language* over A is any subset of A*. Put verbally, a ‘language’ in this abstract sense is some set of ‘legal’ strings from our alphabet A. Put concretely, lets say an alphabet A_1_ consists of the digits 0–9. Then any integer number is a member of a language defined over this alphabet (a member of the language denoted by A_1_*), but the number 1.35 is not (because the ‘.’ is not in our alphabet). The string ‘cat’ is, for the same reason, not a member of this language. Similarly, if our alphabet consists of the letters of the Roman alphabet a–z, we could define some more specific languages where ‘cat’ is contained in the language but ‘katze’ is not.

These terms and their definitions seem quite intuitive and easily understood. The danger is that they are so easy to understand that it is easy to forget what they do and do not involve. Most prominently, there is no discussion of meaning within this framework. Although we can easily design a grammar, in the technical sense, that can accept ‘cat’ while rejecting ‘katze’, this system has no understanding that these strings mean ‘feline animal’, or indeed that they could mean anything at all. A grammar responds purely to sequence and has no way of embodying meaning or of ‘understanding’ the signals that are fed to it. These systems are purely syntactic and have no semantics. Thus, ‘language’ in FLT is missing a substantial component of natural language (the central component of meaning). A ‘language’ in this formal context is a set of strings (a ‘stringset’), and nothing more.

Continuing our exploration of FLT, the simplest way to define a finite language is to simply list all its members. This ‘brute force’ approach is a possibility as long as the language is finite, e.g. for the set containing the words of English (e.g. a dictionary omitting names, loan words or neologisms). It will not, however, be able to deal with the integers, or the sentences of English, because the number of integers or English sentences is unlimited, and the list could never be complete (any candidate finite set can always be ‘trumped’ by adding ‘Mary thinks that’ to a randomly chosen member of the set). If the language in question is infinite, it cannot be listed, and our only hope is to come up with some finite set of rules to generate the language, termed a grammar. A *grammar* is a finite set of rules that specifies some (typically infinite) language. Note that the sentences making up such a set will themselves be finite (in the same way that the set of integers is infinite but each integer is itself finite).^4^

We are finally in a position to restate the Chomsky hierarchy in explicit formal terms. The Chomsky hierarchy incorporates a nested set of automata of increasing power, each of which can generate the strings of some formally defined class of languages. The automata (which have already been discussed) are shown with their corresponding formal language classes, in figure 2. Our old friend, the FSA, is at the centre, with its corresponding language family, the regular languages. Next come the context-free languages, defined by PDAs, which are subsets of context-sensitive languages defined by linear-bounded automata. Finally, in the outer and most powerful ring, we find the languages recognizable by Turing machines. The grammars defining these languages have been given many names, but they were called ‘type 0’ by Chomsky (who dubbed a certain subset of these grammars ‘transformational grammars’ [24]).

A subtle terminological difficulty arises from the nested aspect of the rule systems just described. Because FSAs are contained within the class of PDA, the term ‘pushdown automaton’ *sensu lattu* logically includes all FSAs as a subset or special case. However, these terms are often used informally to exclude their subsets, much as the term ‘reptile’ is used by biologists to delineate all those descendants of the ancestral reptile who are not birds or mammals (the correct term ‘non-avian non-mammalian amniotes’ being a bit of a mouthful). Because this type of usage is quite indispensable in the research programme outlined here, we will explicitly define it. Using basic set theory, we can define any of these automata *sensu strictu* in an exclusive manner. Thus, we could use the term ‘PDA *sensu strictu*’ to delineate all PDAs that are not simply FSAs (in logical terms, this is the set of type 2 grammars omitting the set of type 3 grammars). Of particular importance in the present context are the ‘supra-regular grammars *sensu strictu*’, which we define as the class of all automata (that is, all Turing machines) that are not simply FSAs (that is, type 0, omitting type 3 grammars or equivalently ‘all grammars above the finite-state level’). This is the sense in which the terms ‘supra-regular grammar’ or ‘supra-regular processing’ will be used for the rest of the paper.

### (f) Formal language theory and natural language

As already suggested, Chomsky's primary interest in formalizing automata and organizing in this way was to provide an initial framework for understanding human natural language (‘natural’ meaning languages such as Warlpiri, French or English, as opposed to artificial languages such as mathematics, PROLOG or C++). In particular, Chomsky pointed out that English cannot be captured by a regular or ‘finite-state’ grammar (FSG) because it includes structures (particularly, phrase structures with multiple long-distance dependencies and recursive sentence structures) that are beyond any FSA's capabilities. He further argued that various linguistic phenomena of movement and sentence transformation (e.g. from active to passive) are beyond the capability of context-free languages as well, and thus that natural languages must occupy some broader subset of the type 0 grammars (which he termed ‘transformational’). However, it quickly became clear that transformational grammars are in fact too powerful, requiring a cumbersome set of constraints to make grammars with this degree of power tractable. Subsequently, theoretical linguists working within this formal paradigm have gradually honed in on the level of computational power required for natural language [25,26]. After some years of suspicion that context-free grammars were up to the task, it is now clear that certain phenomena of natural language require context-sensitive grammars, and most researchers in this field now agree that human languages require ‘mildly context-sensitive’ grammars (MSCGs): grammars whose power is just a bit beyond those captureable by a context-free grammar [27–29].

What does it mean to say that ‘natural languages require grammars at the mildly context-sensitive’ level? First, note that any of these abstract classes of automata, including the weaker class of FSAs, contain many automata far beyond that of any human being. For instance, the Manhattan phone book is a finite list, easily captureable in a simple FSA that has one state for each name/number pair, but this language is far beyond the capacity of any human. Thus, the statement that ‘human languages require grammars of at least the power of a finite state automaton’ does not imply that the human brain could instantiate *any* FSA. Similarly, just because some animal species can be shown to do various tasks at the finite-state level, we cannot assume that they can induce *any* FSG. Whatever class of computational systems natural language entails, it will always be some subset of the categories of automata described in FLT (figure 2). Thus, in applying the theory to real organisms, we can use it as a general road map, but we never expect any of these very abstract classes of grammars or languages to be co-extensive with our own capabilities (or those of animals or children).

This approach might correctly be termed ‘syntactocentric’ [30], but exploiting FLT in empirical work in no way denies the central importance of meaning in language. Rather, it reflects an analytical, ‘divide and conquer’ strategy that chooses one component of the vast complex of human language, focusing on form rather than content. Fortunately, this form-based approach has been immensely productive in computer science, underlying many of the technological advances we take for granted today, and thus does not seem too limited to be of interest. More importantly, the current understanding of the most complex signalling systems in other animals (for example, bird or whale ‘song’), along with other rule-governed systems of humans (e.g. music) suggests that they, like formal languages, are focused on structure and *not* complex meaning encoded into units of the signal. The empirical approach we advocate here relies on explicit formal theories as the basis for experimental design, eschewing questions of signal meaning for the time being. Thus, ‘language’ in the formal sense used for the remainder of this paper means simply a set of strings defined by some grammar. No notion of meaning is entailed or implied for the ‘grammars’ we consider: they simply accept or reject strings as belonging to some language.

There are explicit theories that concern the information in signals [31] (although this is quite different from meaning, as emphasized by Shannon [32]), along with semantic models treating meaning in its own right [33,34]. There are also empirical paradigms that focus on the acquisition of meaning by children [35–37] and animals [38]. Finally, it is possible to combine artificial grammar learning with meaning in the laboratory to create ‘artificial language learning’ experiments [39]. There is thus no conflict between a focus on form (syntax or ‘grammar’) and content (meaning or ‘language’)—these are complementary fields of study that must, ultimately, be synergistically combined.

Given this caveat about the purely syntactic nature of FLT, one might ask why anyone should be interested in the question of where human (or animal) capabilities lie in the classification system of FLT. Here are a few reasons, ranging from practical to theoretical. From a purely practical viewpoint, scientists attempting to create computer programs that deal with corpora of data are greatly aided by knowing where the signal-generating system they are studying lies in this system. For instance, the parsing and compiling of either regular or context-free languages are well-defined problems with practical working solutions, but this is not true for context-sensitive languages (*sensu strictu*). The difference between a finite-state and context-free representation is also important to keep in mind when compiling computer code or analysing natural language texts.^5^ Knowing whether such texts demand context-free (or higher) powers, or not, is thus very valuable for anyone interested in building fast, robust computing systems. Similarly, recent years have seen an explosion of interest in complex animal signals such as bird song and whale song, and a vast amount of data have been pouring in from both laboratories and the field that needs to be processed by computer [40]. The tools applied to this task need to be capable, at the formal level, of dealing with the actual complexity of these signals, and if such signals could be shown to require supra-regular grammars, the current crop of finite-state tools typically used to analyse them would be demonstrably inadequate.

In addition to these practical issues, there is a deeper theoretical reason for interest in the formal power of signal-generating systems. The core difference between human natural language and signal-generating systems such as music or birdsong, which can also generate highly complex signals, is that linguistic signals are used to transmit equally complex thoughts from one mind to another. Although music certainly communicates (e.g. mood or emotion, energy and many other powerful and subtle ‘messages’), there is no direct correspondence between the units of music (notes, phrases, etc.) and the structure of thought. For all its power and greatness, music simply cannot communicate plebian facts such as ‘the lion is in the third cave from the right’ or ‘you need to soak that nut in water and ashes for two days before you eat it’, nor can you use a musical phrase to represent those thoughts to yourself. Indeed, if (as in a few isolated cases) we use musical means such as drums or whistles to communicate thoughts, it ceases to be music and becomes language (‘drum talk’ or ‘whistle languages’). Thus, an intimate correspondence between signal and meaning is the *raison d'etre* of language, and the key factor differentiating it from music, and, as far as we know, the diverse signalling systems of every other species on our planet.

There are many grounds for suggesting that thought itself has a tree-like, hierarchical structure [41]. Research in memory, category formation, word learning, visual cognition, Theory of Mind and many other fields all point to this conclusion [42–44], and Herbert Simon has advanced strong theoretical arguments for why a system of thought *must* have such a structure [45]. But if thoughts have a tree-like structure, and are unlimited in number, any signalling system capable of encoding thoughts (that is, any ‘language’ worthy of the name, possessing semantics and thus beyond the rigorous confines of FLT) must be able to capture this structure. This is a potentially deep reason that natural languages have, and arguably must have, hierarchical phrase structure (that is, must go beyond simply stringing items together in a simple finite-state system)—they would be inadequate vehicles for thought if they did not. Although, by introducing meaning, this argument clearly goes beyond FLT, it provides the broader context in which these questions become centrally important to anyone interested in natural language as a whole. This has been clear since the beginnings of the discipline: the so-called ‘weak generative capacity’ (the ability to match stringsets alone) is of quite limited interest. Ultimately, the ability to recover the phrase structure(s) underlying a string (roughly speaking, ‘strong generative capacity’) is much more interesting, and obviously critical for recovering structured thoughts from linear signal strings. If it could be shown that the signal-generating system of some particular species is limited to a simple serial FSG (e.g. the chickadee calls of Hailman & Ficken [15] and Hailman *et al*. [46]), we need wonder no further why that system is not used to express unlimited combinatoric meanings and complex thoughts. Thus, although FLT only gives us tools for exploring signalling systems as stringset, not as meaningful systems, discovering whether the signals generated and processed in animal communication systems are limited to simple finite-state systems or not will have important ramifications for understanding their capacity to convey meaning, and ultimately for understanding the biology and evolution of language.

## 3. The role of infinity in formal language theory

Before we turn to experimental work grounded in FLT, we will briefly discuss the controversial issue of ‘infinity’ in discussions of language. This is an old issue, nicely encapsulated in Wilhelm von Humboldt's suggestions that human language makes ‘infinite use of finite means’ [47]. Clearly, every individual human has a finite memory, a finite lifespan and a finite (though astronomical) number of neurons and synapses. This has always been accepted [3,6,48]. Nonetheless, most linguists or computer scientists happily accept that any natural language such as English or Chinese is infinite (in the sense of ‘unbounded’), in precisely the same way that the set of the integers is infinite. One argument for this parallels the argument for numbers: if someone claims to have identified the largest possible integer, you can easily prove them wrong by simply adding one to their proposed number. In the same way, any proffered ‘longest sentence’ *x* can be trumped by simply generating ‘John thinks that *x*’. Although each of these sentences is of a fixed and a finite length (there are no infinite *sentences*), the *set* of sentences is infinite. From this mathematical perspective, we should no more doubt the infinity of English than we doubt the infinity of the integers.

However, there are more subtle arguments for and against the importance of infinity in natural language [49]. For example, the list of sentences a child hears before fixing on a grammar of English is surely finite, as is the list of all sentences an individual will produce in his/her lifetime. In principle, such lists could be captured by a finite-state system, in the extreme case simply as a list of those sentences. In contrast, all of the proofs used in FLT to demonstrate supra-regularity use the argument of infinity to prove their case (typically this involves invoking the pumping lemma [17]). From a strictly mathematical viewpoint, suspending the axiom that languages are infinite would invalidate most such proofs, and thus greatly weaken FLT.

However, no one supposes that the child simply memorizes all heard sentences: any language user can generate and understand novel sentences, beyond the finite input they received in childhood. *Some* system of more general or abstract rules is necessary to account for this ability. As Chomsky notes [3], ‘a grammar must reflect and explain the ability of a speaker to produce and understand new sentences which may be much longer than any he has previously heard’. The minimum that we might need to account for such generalization is a probabilistic model over a finite-state system (some form or another of a ‘Markov process’). But as Chomsky further observes in the same passage ‘the point is that there are processes of sentence formation that this elementary model for language is intrinsically incapable of handling’, and those include sentences with multiple embedding and nested- or crossed-dependency. Thus, ‘the assumption that languages are infinite is made for the purpose of simplifying the description’ [3], and to allow mathematical proofs that apply to all and every sentence. But there is no theoretical difficulty at all in limiting stack depth or tape length in supra-regular grammars.

Another convincing counter-argument, owing to Levelt [6], goes as follows. Let us assume that human language use *could* be modelled by a FSA, augmented with transition probabilities (a form of Markov process). For this model to have any psychological validity, a child would need enough data to infer these probabilities from the input. So we can ask what order of Markov approximation would be needed for typical sentences. This reduces to the question ‘how many words can separate two words that are dependent upon one another in a sentence?’ In the grammatical English sentence ‘The woman you recently invited to come to New York and give a lecture in our department seems to be sick’, 15 words intervene between the inter-dependent words ‘woman’ and ‘seems’. An attentive English speaker will certainly notice if this pair were incorrectly inflected (e.g. ‘The woman … seem’ or ‘The women … seems’). Hence, we would need a *k*-limited Markov source with *k* = 15 to capture this dependency reliably. But Levelt shows that even with unrealistically lenient assumptions [6, vol. 3, p. 76], such a Markov grammar would require an enormous number of parameters, on the order of 4^15^, or more than one billion. The busy child would need to set about 30 parameters per second, throughout all of childhood, to assimilate such a model. Thus, a finite-state model must be limited to be learnable (and thus unable to deal with long-distance dependencies) *or* it could be theoretically adequate but, owing to the huge number of parameters, practically useless as a model of the child. This argument can be made from many different perspectives, but will always come to the same conclusion: given realistic assumptions, no regular grammar can adequately model English or any other natural language. This is both a practical and a theoretical conclusion.

This combination of arguments has led most commentators to accept the supra-regular hypothesis for humans. Of course, *any* model of human cognition will make simplifications, and thus will be inadequate in certain ways. This is intrinsic to model-building. What we seek are models that make the right generalizations, and that fail in reasonable ways. For example, we can make a simple modification of the weakest supra-regular system, a push-down automaton, in which the stack memory is of a fixed, limited depth. Such a model has no problem with long-distance dependencies, but it *will* have problems with multiple levels of embedding. This is precisely what is observed in humans experimentally, in abundant psycholinguistic research [50,51]. In other words, supra-regular models with finite stores (limited stack depth or tape length) fail in ways that seem much more realistic as models of human performance.

In conclusion, we should not conclude from the importance of infinity in formal mathematical proofs that infinity plays a central role when we turn to practical empirical issues. Infinity is a powerful tool for abstraction, and its judicious use in mathematics allows a kind of certainty that is wonderfully satisfying. For example, Fourier's Theorem *proves* that any complex signal can be built up by a series of sine and cosine waves. Unfortunately, the proof requires an infinitely periodic signal (which continues unvarying from the infinite past to the infinite future), as well as an infinite set of sine waves. Despite these unrealistic assumptions, the discrete Fourier transform, applied to real signals, turns out to be an incredibly powerful tool at the heart of every mobile telephone and spectrographic programme on the planet today. In the same way, we can readily assume that PDA's stack or our Turing machine's tape will be of limited depth, and try to match this to empirical observations of finite humans. Although in doing so we lose the ethereal certainty of theorems, we lose few if any of the practically relevant insights of FLT.

## 4. Empirical investigations using formal language theory

In the rest of this paper, we explore how FLT can be used, practically, by biologists, psychologists and neuroscientists, to design and execute experiments and analyse the resulting data. Often, such studies use artificial grammar learning (AGL) paradigms, see [52]. Because most of the recent literature reviving the supra-regular hypothesis has focused on the finite-state/context-free distinction, we start with a detailed investigation of one particularly simple supra-regular grammar: the ‘counting grammar’ A*^n^*B*^n^*, which has been the focus of numerous recent studies.

### (a) A^n^B^n^: a model supra-regular grammar

The stringset defined by A*^n^*B*^n^*, in which the number of ‘A’ units is precisely matched by the number of ‘B’ units, has played a prominent role in the development of FLT. It is a textbook example of a simple language that cannot be captured by a regular grammar, as already discussed. Despite its ubiquity in the theoretical literature, to our knowledge, the first use of this grammar in experiments was that by Fitch & Hauser [8], who compared the acquisition of two different grammars in two different species: humans and cotton-top tamarins, a New World monkey species. One grammar was the simple regular grammar (AB)*^n^*, which entails any number of ‘AB’ units, and the other was A*^n^*B*^n^*. In both cases, the units were consonant–vowel speech syllables, with the A units spoken by a human female and the B units by a male. Fitch & Hauser found that, while college undergraduates were able to master both grammars, the monkeys only showed above-chance rejection of non-grammatical stimuli for the regular grammar. The monkey's success on the regular grammar showed that the techniques were adequate to elicit rule learning, with generalization, from this species. Fitch & Hauser concluded from this pair of results that monkeys ‘can spontaneously master’ the regular grammar, but are unable to cope with the supra-regular grammar, and thus that ‘tamarins are unable to process a simple phrase structure’ (where ‘phrase structure grammar’ was explicitly defined to mean a supra-regular grammar *sensu strictu*). This conclusion is clearly consistent with the *supra-regular distinctiveness* hypothesis discussed in Fitch *et al*. [52].

Unfortunately, this conclusion was immediately misinterpreted as concerning ‘recursion’, in a commentary in the same issue by David Premack, which stated ‘In a paper on page 377 of this issue, Fitch & Hauser report that tamarin monkeys are not capable of recursion. Although the monkeys learned a non-recursive grammar, they failed to learn a grammar that is recursive. Humans readily learn both.’ [53, p. 318]. This was an unfortunate mischaracterization, because the Fitch & Hauser paper drew no conclusions about, and indeed made no mention of, recursion. Their inference was explicitly focused on the supra-regular boundary, which has no clear relationship to recursion or recursive rules (see below). It was quickly pointed out that there are many ways to recognize the A*^n^*B*^n^* language [54], only some of which might necessarily involve recursion. Unfortunately, the incorrect belief that A*^n^*B*^n^* provides a litmus test for recursion was further perpetuated by a second study testing for recognition of the A*^n^*B*^n^* grammar, this time in starlings. Gentner and co-workers [9] found convincing evidence for recognition of A*^n^*B*^n^* and titled their paper ‘Recursive syntactic pattern learning by songbirds’ (although in the text of this paper, the authors apparently recognize that the actual property being tested is context-freeness). In a commentary on the starling paper, Gary Marcus [55] stated that ‘The A*^n^*B*^n^* language is generally assumed to be recursive’. As a result of these multiple characterizations, there is now considerable confusion in the literature about what, exactly, mastery of A*^n^*B*^n^* (or other supra-regular grammars), by humans or any other species, is supposed to indicate. We now discuss the possibilities.

### (b) Mastery of A^n^B^n^ indicates a supra-regular system

From the viewpoint of FLT, a system's ability to recognize the stringset generated by A*^n^*B*^n^* tells us one thing, and one thing only: that the system is supra-regular (beyond finite state), and therefore has some form of auxiliary working memory, such as a push-down stack, counter or tape, that is not available to an FSA. Although a weak automaton that can recognize A*^n^*B*^n^* is a PDA, with a stack depth limited to the maximum value of *n*, any more powerful automaton (such as a linear-bounded automaton or a Turing machine) can also recognize (or generate) this stringset. Thus, simple mastery of this grammar by some system is not sufficient to tell us *where* in the nested class of systems occupying the supra-regular portion of the Chomsky hierarchy it lies: only that it is supra-regular.

**Figure 3.**
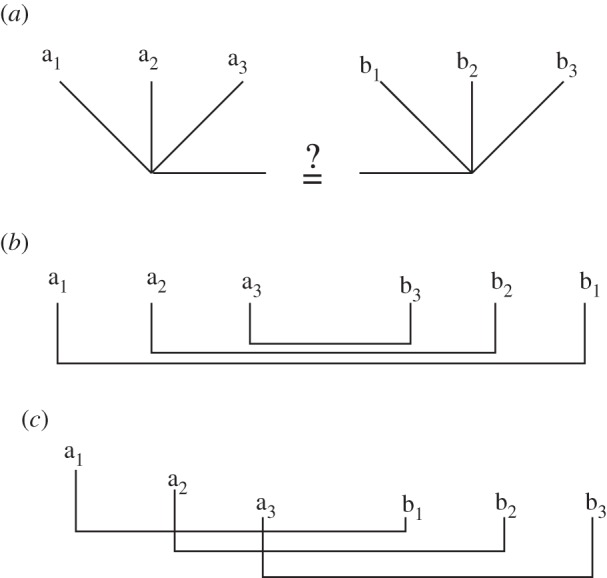
Three possible strategies, and corresponding structures, for recognizing A*^n^*B*^n^*. (*a*) The simplest strategy is ‘count-and-compare’: count the number of ‘a's and the number of ‘b's, and then accept the string if they are equal. This strategy is supra-regular, and generates a single hierarchical level. (*b*) An alternative strategy yields a ‘nested’ or ‘centre-embedded’ structure, and is a natural strategy for a pushdown automaton because it matches each ‘b’ with the most recently seen ‘a’. (*c*) A third strategy yields a ‘crossed’ dependency, and cannot be accomplished with a single pushdown stack. It thus requires at least a context-sensitive grammar.

This ambiguity has important implications for the parsing of A*^n^*B*^n^* strings, as illustrated in the structural diagrams of figure 3. Each of three diagrams exemplifies a different computational mechanism able to recognize the A*^n^*B*^n^* language. The top-most, which is the most obvious and appears to capture what humans spontaneously do when confronted with this stringset, could be called ‘count and compare’. This involves simply tallying the number of ‘A's, storing that number, tallying the number of ‘B's that follow and tallying *that* number and then comparing the two. This could be implemented by an integer register that is incremented by one for each A, and decremented for each B (it should then be 0 for grammatical strings). Two registers could also be used, one holding each of the two counts, and comparing them (figure 3*a*). A PDA could recognize the same stringset by storing the number of ‘A's as a series of marks, pushed one by one onto a stack, and then ‘erased’ by removing them from the stack for each corresponding B. An MCSG compatible solution would be to write a 1 for each A on a tape. Once the B phrase starts, this system would rewind, and then cross off a one for each successive B. In either of the last two cases, an empty stack or a blank tape would be required for acceptance. Crucially, *all of these alternative algorithms require supra-regular processing resources* (whether register, stack or tape), and each suggests a different order of processing. What is relevant then is that there is some way of representing the exact number of ‘A's, and if this value is not bounded *a priori* to any fixed number, the system is perforce supra-regular. No conclusions about recursion are warranted.

### (c) Eliminating finite-state ‘cheats’ with generalization and mis-matched foils

**Figure 4.**
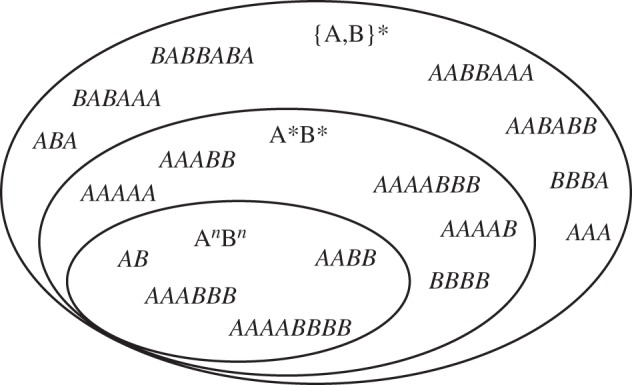
Regular string supersets for A*^n^*B*^n^*^.^ Although recognition of the specific stringset A*^n^*B*^n^* requires a supra-regular grammar, various regular languages contain A*^n^*B*^n^* strings as special cases. For example, the regular language A*B* includes all strings of ‘A's followed by ‘B's, including those where the number happens to be the same. Similarly, the regular language {A,B}* simply means ‘any string of ‘A's and ‘B's’ and also obviously includes A*^n^*B*^n^* strings as a special case. Thus, perhaps non-intuitively, the most inclusive languages (the outer circles of the figure) require less powerful computational machinery.

What evidence is needed for us to conclude that a system ‘recognizes’ the A*^n^*B*^n^* language? As figure 4 illustrates, there are many possible regular grammars that could accept strings of this language, and correctly reject many others, and we need to exclude these alternatives if we wish to infer that our system instantiates a supra-regular grammar [9,54]. For example, the set of all strings made up of ‘A's and ‘B's (written {A,B}*) includes A*^n^*B*^n^* as a subset, as does the set of all strings that start with ‘A's and end with ‘B's (written A*B*). Similarly, the union of two regular grammars, A^2^B^2^ and A^3^B^3^, accepts all A*^n^*B*^n^* strings where *n* = 2 or 3. These and other regular grammars provide potential ‘cheats’ that would allow a regular system above-chance performance in experiments like these.

While it is difficult to exclude all *possible* regular grammars, we can eliminate most of the reasonably simple ones by employing foils (to exclude the overly general cases) and extensions (to exclude the overly specific grammars). Thus, after exposure to A*^n^*B*^n^* strings where *n* = 2 or 3, we can then test our candidate system with A^4^B^4^ (*n* = 4) strings. If the system has induced the supra-regular rule, it should accept these generalizations. In contrast, the ‘regular union’ grammar given earlier would reject such extensions. So this provides one crucial test, allowing us to empirically exclude overly specific regular grammars. Both humans [8] and starlings trained on A^2^B^2^ [9] accept such generalizations, suggesting that neither species implements overly specific templates to identify their stringsets [56].

A second possibility is an overly lenient grammar that accepts the target strings but many others besides. A particularly crucial superset of A*^n^*B*^n^* is the regular language A*B*. A system implementing this grammar would accept all A*^n^*B*^n^* strings and correctly reject ABAB or BAAB strings. The crucial test in this case is the ‘unmatched foil’ A*^n^*B*^m^*, where *n* ≠ *m*. Such strings will be accepted by A*B* or similar variant regular grammars, but clearly rejected by any system implementing A*^n^*B*^n^*. Although Fitch & Hauser did not test for this, several later studies [11,57] showed that humans spontaneously reject such unmatched foils, strongly suggesting that they induce the supra-regular grammar as opposed to A*B*. Starlings also rejected such mismatches [9].

However, there is a third and more subtle possibility, noted by van Heijningen *et al*. [58], that different subjects might implement different regular grammars, and that the composite result (if all individuals are lumped together) might appear to constitute significant evidence for supra-regularity, even if the behaviour of each individual subject is consistent with a simpler set of regular rules. Excluding this hypothesis involves either statistical analysis by individual and/or a maximum-likelihood approach, where each grammar is treated as a hypothesis, and the likelihood that this would generate the observed accept/reject data from one or more birds is calculated. Placing zebra finches in an operant set-up very similar to that of Gentner & co-workers [9], these authors [58] argued that both their birds and Gentner's might be ‘succeeding’ on the task using a motley collection of regular grammars [59]. While it is fair to say that this question remains open, these data opened the door for the most recent study.

Abe & Watanabe [60] used a habituation–dishabituation to probe pattern perception in Bengalese finches (*Lonchura striata domestica*), and again provided evidence for learning of the A*^n^*B*^n^* grammar in this species. In this case, rather than operant training, a mere exposure paradigm was employed, and vocalizations produced to different grammars and their violations were used as a dependent variable. The authors found that listening birds chirped more to novel A*^n^*B*^n^* strings, including novel extensions to *n* = 4. Unfortunately, they do not appear to have tested their finches with ‘unmatched foils’ A*^n^*B*^m^*, where *n* ≠ *m*, and thus we cannot exclude the regular grammar A*B* based on these data. The reason, presumably, is that the authors were focused on a different question: item-wise dependency in relation to ‘centre-embedding’. For further critique of this study, see Beckers *et al*. [61] and ten Cate & Okanoya [59].

### (d) Long-distance dependency versus ‘centre-embedding’

This brings us to a second widespread misconception about the A*^n^*B*^n^* grammar: that it necessarily involves centre-embedded dependency relationships between particular ‘a’ and ‘b’ items [62]. There are two reasons that this assumption is incorrect. First, as clarified earlier, although recognizing A*^n^*B*^n^* requires a supra-regular system, we have no basis for assuming any *particular* supra-regular automaton must be used to do so. While one might suggest that a context-free grammar is the most parsimonious assumption, and therefore that nested matching would be most natural (figure 3*b*), a system that possessed a tape (like a linear bounded automaton or Turing machine) might just as well implement a cross-serial matching as in figure 3*c*. This seems particularly likely in the case of humans because we know that cross-serial dependencies are required in some languages such as Dutch or Swiss German (and we can thus infer that humans possess capabilities above context-free, see earlier text). So if the system did infer dependencies between items, there is no compelling reason to assume that these would be nested rather than crossed.

A more important reason is that most grammars capable of recognizing the A*^n^*B*^n^* language make no demands that particular A items should match particular B items. Indeed, one simple way to write this grammar involves a random selection of A and B terminals (figure 3*a*). A different version that would entail dependencies between specific A and B items seems, in principle, more complicated (figure 3*b*). Put in terms of the various mechanisms discussed earlier, there is no reason for an automaton recognizing A*^n^*B*^n^* to write individual ‘a's or ‘b's to its stack or tape memory: it suffices to simply put any mark (e.g. a 1) for *any* A, and then subsequently count or erase them for each B. It is thus not surprising that humans exposed to A*^n^*B*^n^* strings do not keep track of or notice any particular correspondences, even if the experimenter employed a grammar like figure 3*b* to generate them [62]. Since neither of these two grammars is more correct, it is in no sense a failure if human subjects exposed to strings from figure 3*b* induce the grammar in figure 3*a*, because both are fully adequate grammars for recognizing A*^n^*B*^n^*. The assumption of a centre-embedding item-wise dependency appears to rest on  confusion between phrasal dependency (which A*^n^*B*^n^* obviously has) with item-wise dependency (which it does not necessarily have).

Of course, it remains an interesting question what kinds of dependencies humans (or birds) exposed to A*^n^*B*^n^* strings attend to, or can learn, and a considerable literature has grown up exploring this topic, further discussed by several of the papers in this issue. Several commentators have concluded that the ‘count and compare’ option is not particularly relevant to human language, and so although this potential strategy would be supra-regular, it would be of less interest than the centre-embedded or serially linked options [57,63]. Very briefly, two early studies with humans found that humans exposed to A*^n^*B*^n^* strings generated with item-wise dependencies (as in figure 3*b*) failed to notice these dependencies [57,62]. It is worth noting that Perruchet & Rey [62] employed neither generalization over *n* nor ‘mismatch’ foils, and thus the conclusions they can draw from their study about supra-regularity are weak. In contrast, while the Dutch subjects in de Vries *et al*. [57] did successfully reject such unmatched strings, demonstrating their acquisition of a supra-regular rule, they did not recognize violations of centre-embedding dependencies. This led to the provisional conclusion that subjects in these studies had mastered the stringset using ‘counting’ or some similar strategy, rather than embedding. However, several later studies demonstrate that, given proper training, humans can learn *either* nested or crossed dependencies in an A*^n^*B*^n^* framework [64–67], and Bengalese finches may spontaneously master at least symmetrical centre-embedded dependencies [60]. Thus, all three of the structures in figure 3 can be acquired by human subjects, depending on the conditions.

In summary, humans exposed to A*^n^*B*^n^* stringset spontaneously appear to adopt the simplest strategy—matching the number of ‘A's with that of ‘B's—rather than inferring item-wise dependencies. However, with adequate training, humans can induce grammars over any of the three possible structures in figure 3. We stress that all of these are supra-regular, and that although several of the earlier-mentioned studies have been framed as critiques, they all confirm the basic capacity of humans to master this stringset. At issue, then, is not the supra-regular hypothesis, but the ‘item-wise centre-embedding’ hypothesis. Unfortunately, this is not a hypothesis that the A*^n^*B*^n^* grammar is well suited to test: other supra-regular grammars seem much more suited to address this question. In particular, the mirror grammar (written as *ww*^R^, where *w* represents any string and *R* indicates ‘reversed’) is another supra-regular grammar, recognizable by a context-free grammar, well suited to examine pattern-based centre-embedding. A mirror grammar over {A,B} generates strings such as ABBA, BAAB, BABBAB, etc., in which the right half mirrors the left half (and incidentally contains all of A*^n^*B*^n^* as a subset).

### (e) What is the A^n^B^n^ grammar good for?

Assessing this ongoing debate, it seems reasonable to ask whether a further study of the well-studied A*^n^*B*^n^* language is useful. This of course depends on the questions one is attempting to ask. Those who have employed it with human/animal comparisons have, for the most part, been focused on the ‘supra-regular distinctiveness’ hypothesis, and for this, the A*^n^*B*^n^* grammar is and remains a valid tool [8,9,11,58]. In contrast, most human-only studies have focused on the ‘centre-embedding hypothesis’ and drawn negative conclusions about the relevance of this type of grammar for natural language, at least if humans can recognize its strings via the ‘count and compare’ strategy [57,62,63,68]. The argument in this case is that because natural language does not implement counting of words and comparing across phrases, this computational ability is of little interest in understanding language evolution.

There are two answers to this question. The first is that, if one is focused on the ability to infer grammars beyond the regular or finite-state level, ‘count-and-compare’ is just as squarely beyond this level as is the mirror grammar. Crucially, a substantial animal cognition literature demonstrates that many vertebrates *can* count, exactly, for small integers up to four or five [69–73]: one reason that all of the animal studies discussed earlier used small phrase sizes, of four or below. But recognizing A*^n^*B*^n^* requires more than simple counting: the system must count and compare across phrases. The evidence from animals, thus far, suggests that *this* computation, unlike counting, is difficult or impossible for most tested non-human species. This failure seems very relevant to any detailed analysis of the computational capabilities of different species’ brains.

It is also important to remember that operations that seem intuitively ‘simple’ to us may not be at all simple to other organisms. A good example of this is the detection of bilateral symmetry, which seems so automatic and trivial to us as humans that it might seem to be a very basic and primitive operation. However, considerable research indicates that, at least in those species tested, a generalized notion of bilateral (or mirror) symmetry is not obvious to animals, and indeed may be beyond reach, even with training [74,75]. In FLT, mirror symmetry detection is another computational operation requiring at least a context-free grammar. This capability can be probed with the same A*^n^*B*^n^* or mirror grammars that have been used to generate written or spoken stimuli (Stobbe *et al*. [56] provide more evidence of a limitation to sub-regular visual computation, in pigeons and parrots).

If there is a fundamental computational restriction that prevents most species from accessing even bilateral symmetry or ‘count-and-compare’ strategies, this is surely relevant to these species' inability to acquire the syntax of natural language, which by all accounts require supra-regular capabilities of at least this level of computational power. We certainly encourage the testing of multiple species with many other supra-regular grammars, but as a particularly simple starting point, the A*^n^*B*^n^* grammar seems well suited for testing the ‘supra-regular distinctiveness’ hypothesis.

A variant of this positive answer is provided by multiple brain imaging studies in humans that suggest that the use of the A*^n^*B*^n^* grammar (even if implemented by ‘count-and-compare’) activates different neural processing routines from the (AB)*^n^* and similar regular grammars [11,66,76]. We will discuss these findings, which remain contentious, later, but here it suffices to note that the specific regions engaged are strikingly similar to those activated in natural language syntax tasks, whose relevance to human language cannot be questioned [77,78].

This literature also illustrates a potential pitfall of the A*^n^*B*^n^* grammar. From the viewpoint of FLT, the question about whether a species (or a brain region) can cope with supra-regular stringsets needs to be separated from questions of centre-embedding (which is one of several possible strategies for processing A*^n^*B*^n^*) or recursion (which is not a question that can be answered with this type of experiment). While the A*^n^*B*^n^* grammar is simple and well suited for investigating the basic issue of supra-regularity, those interested in issues of dependency might benefit from branching out to other grammar types. For example, the ‘mirror grammar’ *ww*^R^ is not just supra-regular, but its recognition requires long-distance dependencies between classes, and these dependencies are centre-embedded. In contrast, the ‘copy grammar’ *ww* has cross-serial dependencies, which require a supra-context-free computational capacity (and thus a linear ‘tape’ form of working memory, rather than a push-down stack that can cope with A*^n^*B*^n^* or the mirror grammar). We suggest that pitting the mirror and copy grammars against one another may be more rewarding than continuing to apply A*^n^*B*^n^* to questions it is not the best tool for.

## 5. Variations of testing paradigms: a plethora of choices

One difficulty in comparing results across multiple species, or even across studies with humans, lies in the variation in testing paradigms. Starting with humans, and restricting ourselves to the A*^n^*B*^n^* grammar, stimuli have been presented in the domains of written, spoken or synthesized syllables, have involved explicit training with feedback or virtually no instructions (‘mere exposure’) and have employed explicit yes/no answers (verbally [62], or via a computer interface [8,11,63]). Other AGL studies have used more exotic stimuli, including musical or tactile inputs [79].

Regarding animal experiments, there is so much variability that few fair comparisons can be made across species. One key difference concerns training and reinforcement. Humans readily acquire multiple grammars, including A*^n^*B*^n^*, without training or reinforcement in ‘mere exposure’ paradigms using only positive examples. In contrast, most animal studies have involved tens of thousands of reinforced trials over months or years [9,58,80], although a few studies use spontaneous behaviour (e.g. looking behaviour) to investigate what types of ‘pattern conception’ are used spontaneously, without training [8,60]. Each of these approaches has advantages and shortcomings: for exploring fundamental computational limits, training regimes are superior because a failure after extended training is more convincing. For exploring spontaneous learning or species proclivities, mere exposure and looking time provide more relevant information.

Another important difference between techniques is whether single grammars or dual grammars are presented. In traditional human AGL work, as in spontaneous techniques using animals, positive exemplars from a single grammar are first presented in the exposure phase, and then single-test stimuli are presented to be accepted or rejected [8]. This allows researchers to investigate what is learned from exposure to positive exemplars. In contrast, most training research with feedback requires negative exemplars to be presented as well. So, in a two-alternative forced-choice paradigm, one of the choices must be incorrect and thus stem from some other grammar than the one of interest. Even if the ‘positive’ exemplars (the ones whose choice gives a reward) are from a particular grammar, subjects might be learning to avoid the second grammar. This complicates the design and interpretation of such experiments.

In general, there are too few studies in which different species are given the same stimuli in comparable tasks to permit fair comparisons. The failure of tamarins in a spontaneous task involving A*^n^*B*^n^* [8] cannot be directly compared with the apparent success of starlings on the same grammar [9], given that the starlings received tens of thousands of trials of training with feedback to achieve this success. The best species comparisons available to date are those that use spontaneous or mere exposure techniques to compare humans and animals [8,81] or which provide training to both species [56]. Comparisons across animal species will require collaboration among laboratories, and clear decisions about the hypotheses to be tested and resultant experimental design.

One final issue of comparability is particularly salient in auditory AGL tasks using vocalizations. Fitch & Hauser [8] used recorded human voices to test both monkeys and humans, and it might be that these stimuli are less salient to tamarins than conspecific vocalizations might be (though the monkeys’ success on the (AB)*^n^* task indicates that they do pay attention to patterns in human voices, which is unsurprising given captive animals’ close and dependent relationship to humans for feeding and care). Human voices have also been used successfully with rats [80]. However, most animal studies have used conspecific vocalizations to build up the test strings [9,58,60], giving greater success (but unfortunately have not tested any other species, such as humans, with the same strings). We suggest that in the future, animal researchers test humans with identical strings to allow at least this species comparison. Another way around this problem is to use strings built up of abstract sounds (e.g. synthesized musical sounds), or abstract images [56,82] to allow a fair, neutral comparison among different species.

## 6. Empirical investigations II: neuroscientific data

The second major wave of research capitalizing on the AGL/FLT combination is in neuroscience, and particularly human brain imaging research. An important research paradigm using the FLT framework is the AGL learning paradigm introduced more than 40 years ago by Reber [83]. In these pioneering behavioural studies, Reber used AGL to demonstrate that humans can implicitly learn rule systems consisting of a set of non-linguistic rules governing the concatenation of meaningless letter strings. In this work, FSGs were used as a model rule system, simple enough to learn, but complex enough to be challenging. But the focus in this literature was on the learning and its implicit/explicit nature rather than on the grammar itself, and despite scores of publications this research paradigm apparently never ventured beyond regular grammars [84–86].

In contrast, a new and fast-growing field has used FLT to design many different grammars, including supra-regular grammars, to probe the neural mechanisms that underlie abstract pattern recognition abilities in humans, and compare them with those involved in natural language processing. Such studies involve brain imaging technologies such as electroencephalography (EEG) and magnetic resonance imaging (MRI). EEG measures brain electrical activity, while functional MRI (fMRI) images blood flow. Both techniques thus index cognitive function in the brain. Transcranial magnetic stimulation (TMS) uses a powerful magnetic field to perturb neural function, allowing experimental evaluation of the role of particular brain areas. Finally, diffusion-weighted MRI or diffusion-tensor imaging (DTI) can be used to image both grey matter anatomy and the white matter fibre tracts connecting different brain regions that constitute a network. A crucial aspect of the new brain imaging studies lies in the comparison of the brain activation for artificial and natural grammars to investigate whether particular types of artificial grammar recruit brain regions and networks used in processing natural language grammar.

### (a) Natural language data

One focus of particularly intensive, and controversial, research in recent neurolinguistics concerns Broca's area, or the left lateral prefrontal cortex more generally, and its role in processing linguistic syntax and sequential patterns. The term ‘Broca's area’ refers anatomically to the pars opercularis and pars triangularis in the left inferior frontal gyrus (LIFG) and cytoarchitectonically to Brodmann's areas BA 44 and 45 [87,88]. In Broca's original article, this area was seen as a speech production centre, but seminal research from Caramazza, Zurif and co-workers revealed that this same region plays an important role in syntax processing during comprehension as well [89]. This has emerged as an extremely robust finding in the neuroscience of language [90,91], but consensus concerning its exact significance remains elusive [92–99].

The debate concerns the degree to which Broca's area is specialized for syntax processing or even linguistic processing more generally, or rather subserves some domain- and modality-general computations such as hierarchical planning, working memory or selection among competing alternatives. Advocates of different models often have different theoretical backgrounds, and adjudication is made difficult by the theory-specific characterization of each different model. One of the first models [92] assigned the computational role of Broca's area to a particular form of syntactic working memory needed to process syntactically complex sentences, thereby specifically relevant for syntactic processing.

In a related, but more specifically linguistic, model, Grodzinsky [93] suggested a syntax-specific role for Broca's area, based on the traditional generative notion of ‘syntactic movement’. ‘Movement’ refers to a particular syntactic computation that can be described as follows. Complex sentence structures often feature words that make reference to distant ‘empty’ slots in the same sentence, and a traditional approach to such long-distance dependencies is that they result from an abstract ‘movement’ of the word away from its original location. Oversimplifying for clarity, if we start with the sentence ‘John likes sandwiches’, we might construct the interrogative ‘What does John like?’ by changing ‘sandwiches’ to ‘What’ and then moving this word to the front of the sentence. Thus, the interrogative *wh-* word ‘what’ is linked to the empty slot at the end of the sentence, where the direct object of ‘like’ would normally go (this is thus termed ‘*wh*- movement’). Grodzinsky & Santi [100] reviewed data from aphasic patients, suggesting that only sentences that possess this type of linkage are particularly difficult for Broca's aphasics, and thus that the role of Broca's area is best characterized by the computation of ‘movement’. Using fMRI, they provided additional evidence in support of this view [101].

A neuroanatomically more fine-grained model suggested by Friederici [94] argues for a functional subdivision of Broca's area into an anterior part (BA 47/45) responsible for semantic relations, and a posterior part (BA 44) subserving processing of syntactic relations, in particular long-distance dependencies involving syntactic transformations [102]. A recent overview [91] of fMRI studies on syntactic complexity in different natural languages and different sentence structures including ‘movement’, ‘scrambling’^6^ and ‘nesting’ revealed a clear involvement of Broca's area as syntactic complexity in these constructions increases. Across these different studies, ‘movement’-related activation is localized in the more anterior-ventral part of Broca's area (BA 45), whereas ‘scrambling’ clusters in the more posterior part of Broca's area (BA 44). ‘Nesting’, i.e. the processing of centre-embedded structures, was only investigated in a few fMRI studies. One study that evaluated the processing of centre-embedded sentences found activation in BA 44 increased with the number of embeddings [103]. The three models discussed so far [92–94] all focus on multiple long-distance dependencies, which—as we have seen—typically entail supra-regular processing resources (e.g. a register, stack or tape).

These three models posit an exclusive role for at least some portion of Broca's area in processing syntax. An alternative framework has been suggested by Hagoort [95], who offered a broader characterization of the role of the LIFG as subserving linguistic unification, applying across the domains of phonology, syntax and semantics. ‘Unification’ is a powerful computational operation in which pairs of structures can be combined repeatedly to form larger structures, if certain matching conditions are met. Linguistic unification typically starts by combining lexical items, which have certain necessary syntactic properties (e.g. some verbs may require both a subject and an object, and the verb phrase formed by unifying all these could later be used in a larger construction). Unification is a core operation in many modern grammatical formalisms (e.g. categorial grammar and tree-adjoining grammar) as well as in programming languages such as Prolog [28,104,105]. Unification, in general, requires supra-regular computational resources [27].

Thus, all of these models have in common that they attempt to single out a certain component of syntactic complexity for which Broca's area plays an important processing role. In each case, sophisticated models have been proposed to ground the neuroscientific results in linguistic theory, but in each case the terms used are specific to a given theoretical framework (even though they can cope with the same syntactic phenomena). Progress in testing these different ideas requires an over-arching framework broad enough to encompass all of these possibilities, and precise enough to specify their differences from one another and from other hypotheses about the computational role of Broca's area. FLT, and the theory of computation more generally, seems well suited to this role. In particular, it is noteworthy that *all* of the earlier-mentioned characterizations of the role of LIFG share the necessity of supra-regular processing resources.

### (b) Artificial grammar-learning data

Neuroimaging studies on AGL have tried to elucidate the neural mechanisms of language learning and have provided insight into the nature of knowledge that is learnt in artificial grammars of a given type. Different studies have focused on different aspects of rule and grammar learning.

The first generation of AGL neuroimaging studies focused on the neural correlates of implicit leaning using Reber-type FSGs. They revealed that two aspects of learning, namely similarity-based learning and abstract rule learning, were correlated with two different brain systems. The former learning type was seen to involve the left hippocampus, whereas the latter was found to activate anterior prefrontal cortices bilaterally [106]. Other AGL studies reported activation in the anterior part of the middle frontal gyrus and the parietal lobe bilaterally [107]. Although the term ‘grammar’ was used, these studies focused on the learning of rule-based sequences rather than on language processing. And indeed, the brain activation patterns reported in these studies were somewhat different from the neural network for natural language processing, which usually recruits the left temporal cortex and left inferior frontal cortex [91,108]. Also, event-related brain potential studies reported different patterns for the processing of linguistic and non-linguistic artificial grammars. Violations in non-linguistic sequences (i.e. Reber-type grammars) revealed a domain-general centro-parietally distributed positivity around 300 ms [109], called P300 and considered to be domain-general [110]. In contrast, learners of a linguistic artificial grammar (i.e. mimicking the phrase structure of natural languages) demonstrated an early anterior negativity to violations in the string [111], similar to syntactic violations in natural languages [112–116].

**Figure 5.**
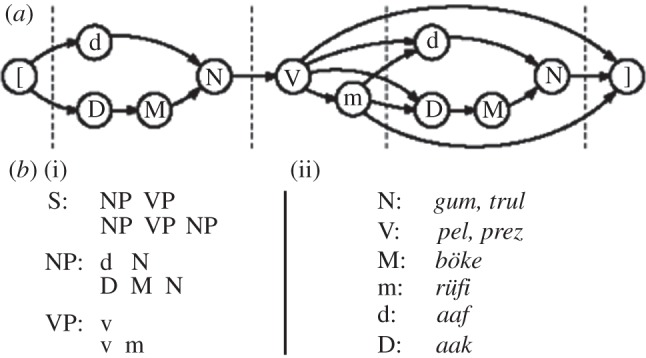
Artificial grammar of BROCANTO. (*a*) Transition from the left to the right following the arrows generates a sentence. For example, {dN Vm DMN}, {dDMN v} and {dN v dN} are correct sentences generated from this automaton. The nodes represent syntactic categories: N (noun), d and D (determiner), v (verb), M (adjective) and m (adverb), and ‘[’ and ‘]’ represents the start and end symbols. (*b*) The rewriting rules (i) of the grammar. The rules define the derivation of S (sentence), NP (noun phrase) and VP (verb phrase) from the terminal symbols given as a set of novel vocabulary (ii).

**Figure 6.**
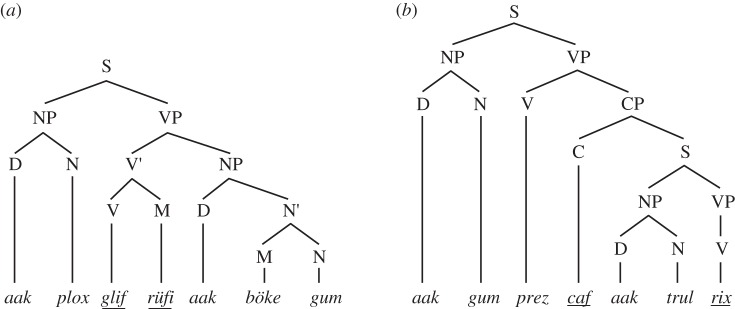
Phrase structures for modified version of BROCANTO. BROCANTO was modified to investigate the difference between grammars that have long-distance dependency (indicated by underlined element (*a*,*b*)) and those that do not. Moreover, the introduction of the complementizer required a word order change in the subordinate clause: from (*a*) verb second position in the main clause to (*b*) verb final position in the subordinate clause. (*a*) Structure with local dependencies. Dependent elements are underlined. (*b*) Structure with long-distance dependencies. Dependent elements are underlined. A set of rewriting rules builds a hierarchical structure. The rewriting rules are represented as binary branches, e.g. S → NP VP. S, sentence; NP, noun phrase; VP, verb phrase; CP, complementizer phrase; D, determiner; A, adjective; N, noun; V, verb; M, verb modifier; C, complementizer.

The second generation of neuroimaging studies tried to relate AGL research more to natural language research, testing artificial grammars more similar to natural grammars, for example, a grammar named BROCANTO [111,117,118] (figure 5). BROCANTO is a simple FSG with a restricted number of syntactic rules and a restricted number of words in different syntactic word classes (e.g. nouns, verbs, etc.). Using this type of grammar, an fMRI study found that the initial phase of learning was correlated with high activation in the left hippocampus known to support item memory consolidation, but that the activation in the left hippocampus decreased and activation in the left Broca's area increased as learning progressed. This learning-related change in the brain activity was interpreted to reflect a transition from similarity-based learning supported by the hippocampus to a syntactic rule-based processing in Broca's area [118]. A follow-up study used a variant of BROCANTO (figure 6, introducing a complementizer that obligatorily required a word order change) to investigate local and long-distance dependencies in a sentence. While the processing of a dependency in a local phrase structure activated the left ventral premotor cortex (BA 6), the processing of a long-distance dependency in a hierarchical structure activated Broca's area (BA 44) [119], indicating a difference in the functional neuroanatomy of these two dependency types. Other studies have used ‘jabberwocky’ sentences to investigate grammatical processing [99,120,121] and found that the network centred on Broca's area was sensitive to grammatical hierarchy, irrespective of whether meaningful or meaningless words were used [99,121].

Another interesting fMRI study compared the learning of a natural and an unnatural language [122]. Italian natives had to learn Japanese or a language with Japanese words but a syntax disobeying the principles of any natural grammar. For the language that followed the universal principles of natural grammars (Japanese), an increase in activation in the left Broca's area and the right prefrontal was found, but not for the language disobeying such rules. This study suggested that artificial grammars following the principles of natural grammars recruit Broca's area (BA 44/45), while artificial grammars disobeying these principles do not.

An early neuroscientific study explicitly adopting an FLT framework, published by Friederici *et al*. [11], contrasted the supra-regular A*^n^*B*^n^* grammar with the regular (AB)*^n^* grammar in a between-subjects fMRI design. They found increased activation of the frontal operculum (an area immediately ventral to Broca's area) for syllable sequences of both grammars, suggesting a role in immediate sequencing. Additional activation of BA 44, in the heart of Broca's area, occurred only with the supra-regular A*^n^*B*^n^* grammar. They concluded by hypothesizing that BA 44 is particularly activated in tasks requiring ‘the computation of hierarchical dependencies’. In a further important finding, this study used DTI to examine the white-matter connections stemming from these two different areas, and uncovered two separable neural networks. While the frontal operculum had preferential connections to the anterior temporal lobe via the uncinate fasiculus, BA 44 connected via an independent dorsally located white matter fibre tract to the posterior and middle temporal lobe: the Broca-to-Wernicke connection via the superior longitudinal fasciculus and the arcuate fasiculus. For further discussion of these tracts see later text.

This paper prompted several further studies working with the same grammar in different ways. One critique suggested that, by using a simple A*^n^*B*^n^* grammar with no item-wise dependencies, these experiments were not getting at the linguistically interesting aspect of centre-embedding [57,62]. As discussed earlier, this argument conflates two different issues that FLT neatly separates: recognition of a supra-regular string-set (which can be achieved in various ways, including the supra-regular ‘count and compare’ strategy), and the structures inferred during string parsing (which might involve simple phrasal chunks, or centre-embedded or cross-serial item dependencies; figure 3). For researchers interested in demonstrating supra-regular computational systems, which of these particular strategies is chosen is irrelevant, because they all go beyond the capabilities of a finite-state system.

Nonetheless, when subjects master the A*^n^*B*^n^* language, it remains interesting to investigate which strategies are used under what circumstances. A considerable literature has now developed that explores this question in detail, often including a brain imaging component [57,62,63,66,68,76,103]. The literature has asked whether, after exposure to A*^n^*B*^n^* strings with predictive dependencies (e.g. A_1_A_2_B_2_B_1_), subjects notice a violation of this dependency (e.g. rejecting strings where the A*^n^*B*^n^* rule is obeyed, but the dependency is violated, e.g. A_1_A_2_B_1_B_2_). After initial results suggesting that ‘mere exposure’ to centre-embedded dependencies in an A*^n^*B*^n^* grammar is not enough for subjects to recognize violations of those dependencies [57,62,63], it has become clear that human subjects *can* learn such centre-embedding, and extend it correctly, but that this takes additional training, exposure or prosodic structure in the training stage [66,123,124]. Several laboratories have now produced convincing demonstrations that humans can learn both nested and crossed dependencies [64,66]. Intriguingly, processing of such dependencies appears to engage the inferior frontal gyrus, and a TMS study showed that this area plays a causal role in such processing [125].

An fMRI study designed to force the processing of centre-embedded relations (e.g. A_1_A_2_B_2_B_1_ whereby A_1_ and B_1_ do not represent particular items, but a class of items) again showed activation of Broca's area (BA 44 in particular) together with motor cortical (SMA) and subcortical areas (basal ganglia) for the processing of A*^n^*B*^n^* grammar sequences compared with (AB)*^n^* sequences [66]. Thus, fMRI studies indicate that both predictive and non-predictive stringset activate Broca's area (BA 44 in particular). Finally, although this literature has primarily focused on centre-embedding, it is also of interest to ask whether humans can learn cross-serial dependencies between items (e.g. with an exposure set involving A_1_A_2_B_1_B_2_ dependencies [64]). As Uddén & Bahlmann [65] review later in this issue, the answer to this question is positive.

Other recent fMRI studies using AGL further investigated Reber grammars, challenging the view that Broca's area is particularly involved in processing non-adjacent dependencies. These studies reported activation in BA 44/45 for a regular grammar learning and classification task [125–127], but also additional large activations in parietal, occipital and temporal brain regions. The finding that simple right-linear grammars activate Broca's area challenges the view that this area is specifically involved in the processing of non-adjacent, higher order hierarchical dependencies [128]. Rather, on the basis of these latter fMRI studies, Petersson and co-workers suggested that Broca's area is ‘a generic on-line structured sequence processor active at different levels depending on the processing complexity’ [126,128]. It remains to be resolved how this generic hypothesis can account for the different activations observed in (AB)*^n^* and A*^n^*B*^n^* grammars, given that these two grammars have the same number of very similar rules [22].

Thus, the combined data from the fMRI studies in artificial grammar learning and from non-language domains suggest that Broca's area supports the processing of structured sequences, and of supra-regular sequences in particular.

Although the literature reviewed earlier illustrates the value of FLT in designing neurolinguistic experiments, we need to separate the question of whether a species (or a brain region) can cope with supra-regular stringset, from questions of centre-embedding (which is one of several possible strategies for processing A*^n^*B*^n^*) and recursion (which, for reasons already clarified, is not a question that can be answered with this type of grammar). While the A*^n^*B*^n^* grammar is simple and well suited for investigating the basic issue of supra-regularity, those interested in issues of dependency might benefit from exploring other artificial grammar types including, for example, the ‘mirror grammar’ *ww*^R^. This not just supra-regular, but its recognition *requires* long-distance dependencies between classes, and these dependencies are centre-embedded. But the other way to approach this issue is to examine particular sentence structures in natural language.

### (c) Brain activation overlaps in natural language and artificial grammar learning

With respect to the identification of the brain basis of supra-regular stringset processing and the possible underlying processing strategies, a direct comparison between AGL and natural language processing may be useful.

Such studies shed light on possible strategies underlying the processing of A*^n^*B*^n^* in artificial grammar by directly comparing it with the processing of centre-embedded structures in natural language. In natural language, the respective long-distance centre-embedded dependencies are not dependencies in a symmetrical structure (as in A*^n^*B*^n^* [11]), but in an asymmetrical structure (i.e. the relation between subject noun phrase and the verb [103]). In the case of natural language processing, a multi-layered hierarchical dependency structure must be computed to achieve understanding, whereas for the simple artificial A*^n^*B*^n^* structure this is not necessary [57,62]. Thus, the observed overlap in the activation in Broca's area for A*^n^*B*^n^* in AGL [11] and in natural grammar processing [103] suggests that humans build up structural hierarchies (even if unnecessary) when dealing with artificial A*^n^*B*^n^* structures.

One additional issue needs to be considered when comparing AGL and natural language. In the AGL paradigm, *novel* rules must be learned, whereas linguistic studies examine pre-existing rules from native language processing. In most AGL studies, performance is only 70–80% correct, indicating that the learned rules are not well established. Performance by native speakers in natural language experiments is usually much higher. The performance in AGL experiments rather is more comparable to the performance level to language learners, be it in first language (L1) or second language (L2) acquisition. The brain activation pattern observed for L1 learners [129] and L2 learners [130] who are not yet proficient usually involves not only Broca's area, but large portions of the entire left prefrontal cortex even for the processing of local dependencies (i.e. violations in a prepositional phrase).

This broad activation pattern bears some similarity to the activation reported for some recent AGL experiments [78,127], where in addition to Broca's area, large portions of the prefrontal cortex (BA 6,8,9,46,47) including the frontal operculum and the anterior insula, and regions in the parietal, temporal and occipital cortices were activated (when contrasted against a low-level baseline). One important factor may be that in implicit AGL paradigms, participants are confronted with a novel judgement task immediately before entering the scanner, whether a grammaticality judgement [78] or preference judgement [77]. This may lead to attention-induced control processes and to an activation of large swathes of frontal cortex during classification, even for a simple Reber-type grammar. This argument accords with the view that prefrontal cortex, including Broca's area, is recruited for processes requiring a high degree of cognitive control [131]. Such controlled processes come into play during L2 processing [130] and during language acquisition [129]. In contrast, they are not necessarily activated during highly trained, automatic processes used in processing native language in the adult brain, or for highly trained processes during AGL. A clear separation in the brain activation for adjacent versus long-distance hierarchical dependencies may thus only be observable in a fully established, mature system.

### (d) Diffusion-tensor imaging: a new perspective

As already mentioned, one study used an MRI technique that allows one to make images of white matter fibre tracts [11]. In this study, probabilistic fibre tracking was used, placing starting ‘seed’ points located in the centre of the functional activation for the two grammars (frontal operculum and BA 44, for regular and supra-regular grammars, respectively). The analysis revealed two different fibre tracts: a ventral system connecting the frontal operculum to the anterior temporal cortex via a ventrally located fibre tract, and a dorsal system connecting BA 44 to the posterior temporal cortex via a dorsally located fibre connection, including the superior longitudinal fasciculus and the arcuate fasciculus. These data were taken to suggest two different neural networks, with the dorsal network supporting the processing of hierarchically structured sequences (for a similar functional view [132]).

**Figure 7.**
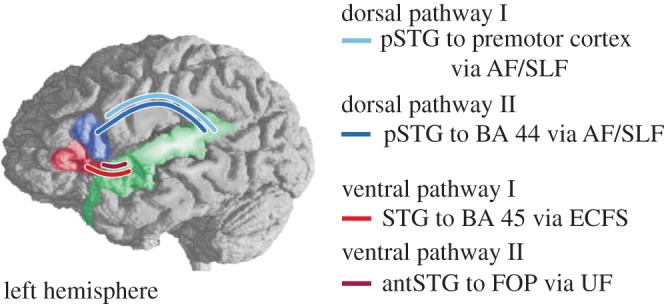
Structural connectivities between the language cortices. Schematic of two dorsal pathways and two ventral pathways. Dorsal pathway I connects the superior temporal gyrus (STG) to the premotor cortex via the arcuate fasiculus (AF) and the superior longitudinal fasiculus (SLF). Dorsal pathway II connects the STG to BA 44 via the AF/SLF. Ventral pathway I connects BA 45 and the temporal cortex via the extreme capsule fibre system (ECFS). Ventral pathway II connects the frontal operculum (FOP) and the anterior temporal STG/STS via the uncinate fasciculus (UF). Reproduced from Friederici [91].

Despite a long history of associating the arcuate fasciulus with language [133,134], an ongoing dispute concerns the precise function of the dorsal pathway [135–137]. A number of researchers proposed that the dorsal pathway supports sensory-to-motor mapping [108,138], and provided good evidence supporting this conclusion. This controversy may be resolved in the light of a recent finding that indicates that in the adult brain, two *different* dorsal pathways can be distinguished: one connecting the temporal cortex and the premotor cortex (possibly supporting sensory-to-motor mapping) and one connecting the temporal cortex and BA 44 (possibly involved syntactic processing) [139,140] (figure 7).

With respect to the ventral pathway, we may also have to consider two functionally separable fibre tracts. There is large agreement that the ventral pathway connecting BA 45/47 to the middle temporal cortex via the extreme capsule fibre system supports semantic processes [108,132,138,141], but it is still open to what extent a ventrally located fibre tract connecting the frontal operculum to the temporal cortex supports the processing of regular structures [11,142]. Unfortunately, fibre tract results can inform us only indirectly about their potential function as they provide only structural information. Connectivity studies do, however, invite a more serious consideration of neural circuits rather than isolated brain regions [141].

### (e) Evolution and development of brain connectivity

A useful perspective on the human connectivity data for regular and supra-regular grammars may be provided by adopting phylogenetic and ontogenetic viewpoints. Phylogenetically, recent DTI data show that non-human primates differ from humans in their connectivity pattern. Comparing macaques, chimpanzees and humans, Rilling *et al*. [143] found that the dorsal fibre tract, from Broca's area or its homologue to temporal cortex, gains in strength from macaques to chimpanzees to humans. This difference seems to be of particular interest in the context of the behavioural finding that humans, but not tamarin monkeys, are able to acquire an A*^n^*B*^n^* grammar [8]. This raises this possibility that a robust dorsal fibre tract connecting BA 44 to the temporal lobe is necessary for supra-regular processing, whether artificial or natural.

A complementary perspective is provided by studies of human populations that have problems with the processing of syntactically complex structures in natural language, such as patients with brain lesions [140], or children who still have not reached adult-like performance [144]. Children who still have considerable problems in processing object-first sentences, i.e. sentences in which the object noun phrase is moved to the front of the sentence, have a significantly weaker dorsal fibre tract than adults, while their ventral fibre system is equally strong [144]. This supports the hypothesis that the dorsal tract is functionally relevant for the processing of syntactic hierarchies. Moreover, it is interesting to note that this particular fibre tract (BA 44 to temporal cortex) is not yet myelinated (and thus not propagating information efficiently) at birth, whereas the ventral system and the dorsal tract from the premotor to the temporal cortex is already myelinated at birth [139].

If the functional interpretation of this latter fibre tract as part of the auditory-to-motor mapping system is correct, we would predict that very young infants should be able to recognize simple regularities in the auditory input. There is good evidence that this is indeed the case, for both AGL [145] and natural language [146]. So far, however, it is not clear to what extent this ability is solely based on the dorsal auditory-to-motor pathway or partly also relies on the ventral pathway present at birth.

## 7. The future

Although the marriage of AGL and FLT has already produced some new and important results, in both the animal and neural domains, this research programme remains in its preliminary stages. Current empirical research provides a detailed exploration of only two particular grammars: the complicated regular grammar introduced by Reber [83] and since explored by scores of researchers, and the supra-regular A*^n^*B*^n^* grammar introduced by Fitch & Hauser [8] and since explored by at least six different research groups. However, there are many other grammars, and empirical approaches, worthy of exploration, and we welcome this ongoing broadening of the field. Investigations of FSGs such as edge grammars [147,148] and other subregular grammars [16] may provide a more detailed dissection of computational primitives particularly relevant to animal researchers [59]. For humans, as discussed already, detailed exploration of other simple context-free grammars such as the mirror grammar or copy grammar will probe the limits of our own pattern-discovery abilities. Innovative experimental designs will play an important role in this—for example, using serial-reaction time tasks and cross-modal auditory/visual AGL [67].

Two further directions immediately beckon. FLT has developed considerably in the last decades in two important directions, both of which offer rich opportunities for empirical research in the ‘Grammarama’ tradition [52]. The first are tree grammars and tree automata, which have been an area of important recent progress in theory [18,149,150] and are beginning to be used as practical models in psycholinguistics [104,151,152]. The tree-adjoining automata of Aravind Joshi require grammars at the mildly context-sensitive level, and thus are computationally equivalent to other linguistic theories such as combinatory categorical grammar, or minimalist grammars, that converge at this level [27,29]. Nonetheless, tree grammars can lead to a rather different view of the nature of computational primitives from those provided by the traditional string set approach, potentially closer to biological and cognitive reality [153].

The second major advance in FLT concerns probabilistic models of syntax [154,155]. In such models, the apparatus of FLT (typically finite-state or context-free grammars) is augmented by calculating a probability for each production or sub-tree [16]. Although symbolic models and probabilistic or statistical models are often contrasted by cognitive scientists, there is a growing realization that there is no conflict between these approaches (indeed, traditional rule-based approaches are just a special case of probabilistic models, but where the probabilities are either 0 or 1). Owing to the need for large amounts of data to calculate the required probabilities, such models initially found powerful application in the domain of corpus linguistics [156]. Applying such models to experimental data will require large samples as well, and thus is appropriate for analysing the results of operant AGL experiments [58]. As web-based experiments become more widespread [157], we can anticipate a flood of data from human participants, testing many different grammars with thousands of subjects, that would be well suited to fitting by statistical models.

In the longer term, we can anticipate that this research programme will be able to replace the highly stylized models of computation used in FLT with more biologically grounded computational primitives [158]. While we strongly support moves in this direction, it is crucial that they be grounded in empirical data rather than in intuitions or assumptions about what computational operations are or are not ‘primitive’. For instance, it seems intuitive to humans that symmetry recognition should be a very simple and basic operation, and thus part of the conceptual toolkit of most visually-sophisticated organisms. Years of pigeon research demonstrates that this assumption is incorrect, and that generalized bilateral symmetry is in fact an extremely difficult concept for these animals to attain [74]. A similar point can be made about the difficulty of cross-serial versus nested dependencies in human experiments: while most people's robust intuition is that the former should be much easier, what few experimental data is available paint a murkier story.

FLT both allows us to characterize such experiments in more general terms (e.g. that generalized symmetry requires a supra-regular grammar) and provides an explicit language for notating and reporting different string types or grammars (as in (AB)*^n^* or A*^n^*B*^n^*). FLT also, and more controversially, provides a set of ‘fixed points’ of computational complexity, such as push-down stacks versus the endless tape of Turing machines, that represent abstract representations of different types of working memory. While future research may show that such abstractions are too artificial to usefully characterize neural computational primitives, performing experiments using them will be the surest and fastest way to find out. Currently available alternative models of neural or ‘natural’ computation [159–162] offer nothing like the scope and specificity, nor the broad acceptance across scientific disciplines, of the theory of computation as treated by FLT.

Thus, we conclude that the empirical research programme combining FLT with AGL will continue, and will continue to be exciting and controversial. Such research will be particularly valuable in uncovering relevant differences among species, and in helping us to characterize the neural mechanisms underlying these differences. This research programme can help itself to a well-developed pre-existing mathematical/computational framework and a rich body of formal understanding and important theorems. If such research eventually leads to a replacement of current formalisms by a computational theory more firmly grounded in biological and neuroscientific reality, it and thus paves the way for its own demise, it will be a welcome sign of scientific progress.

## Endnotes

1 The equivalent of the ‘*’ character of *ls* or *dir* (which borrows its symbols from an earlier regular expression system called *glob*) is actually ‘.’ in grep and ‘*’ has a different meaning.

2 Because any alphabet can be encoded in binary, we can think of these symbols as being limited to ones and zeros without loss of generality.

3 In formal language theory, the ‘*’ operator, known as a Kleene star, means ‘repeat the preceding symbol zero or more times’, and thus *A** means ‘repeat any of the symbols from the alphabet *A* zero or more times’.

4 Note that the number of possible grammars is infinite, but because the number of rules is by definition finite, it is countably infinite (mapping on to the integers).  Unfortunately, the number of possible languages is uncountably infinite (mapping onto the real numbers), and this means that there are many possible languages that cannot be captured by a grammar.  This might seem like bad news until we realize that all of these ‘uncapturable languages’ are in some sense trivial assemblages, simple lists that follow no rules.  If the language in question obeys any rules, then it can be captured by some grammar.

5 A good example is provided by the UNIX compiler-building tools, *lex* and *yacc,* which act as a symbiotic pair: *lex* is a FSA capable of parsing regular expressions, typically used to build a lexicalizer or tokenizer to pull out predefined ‘words’ from a stream of text; *yacc* (‘Yet Another Compiler-Compiler’) is a tool to build context-free parsers.  Because of their higher computational power, *yacc* parsers can easily do things that *lex* parsers cannot (e.g. parse if–then statements, nested loops and recursive parenthetical statements, etc).  The price paid is in speed: although *yaac* could in principle tokenize, it would be a hopelessly slow way to do so, while *lex* tokenizers are guaranteed to operate at very nearly the optimal speed possible.  Thus, the two tools live in happy symbiosis—perhaps this provides a suitable metaphor for some cognitive subsystems as well?

6 ‘Scrambling’ refers to noun phrase displacement in the middle field of the sentence in languages with free word order, such as German or Japanese.
